# Harvest of Red-Legged Partridge in Central Spain

**DOI:** 10.1002/jwmg.391

**Published:** 2012-04-20

**Authors:** Silvia Díaz-Fernández, Javier Viñuela, Beatriz Arroyo

**Affiliations:** Instituto de Investigación en Recursos Cinegéticos, IREC-CSIC-UCLM-JCCMRonda de Toledo s/n, 13005—Ciudad Real, Spain

**Keywords:** *Alectoris rufa*, central Spain, farm-reared partridges, harvest, hunt, intensification, restocking, small game

## Abstract

A basic rule to attain sustainable use of harvested resources is to adjust take to availability. Populations of red-legged partridges in Spain have decreased in recent decades, and releases of farm-bred partridges to improve short-term availability are increasingly common. We used questionnaires and bird surveys to assess whether harvest was related to availability of wild partridges or intensity of farm-bred partridge releases. We studied 50 hunting estates, including 6 administratively labeled as intensive (with few numerical and temporal restrictions to releases). In addition, we considered hunting pressure (number of hunters × hunting days/km^2^) and habitat as explanatory variables in the analyses. In intensive estates, annual harvest was exclusively related to release intensity, indicating that in these estates hunting is detached from natural resources and approaches an industrial activity based on external inputs. In non-intensive estates, harvest was affected by wild stock availability, walked-up shooting pressure, and habitat (greater harvest in estates with more Mediterranean shrubland). In these estates, releases did not increase annual harvest, and can be considered an inefficient practice. Additionally, the relationship between abundance estimates and harvest disappeared in estates with low partridge abundance estimates, suggesting possibilities for overharvesting in a large proportion of estates. Increasing the abundance of wild red-legged partridge through techniques like habitat management, and improving the adjustment of harvest to availability, may be a good strategy to increase long-term harvest in non-intensive estates. Additionally, Government and managers must create ways to segregate and label the estates where only wild red-legged partridges are managed from those where releases are used, to reduce ecological costs of management decisions. © 2012 The Wildlife Society.

Adequate management of natural resources requires a balance between the needs of the public and the long-term maintenance of those resources. A basic rule to attain sustainable use of harvested resources is to adjust take to availability. Simulation techniques like management strategy evaluations (MSE; Punt and Donovan 2007) have shown how decreasing the uncertainty in estimates of fish population size enables a better adjustment between take and availability, contributing to increased yield stability and profitability (Holland 2010). This may be also valid for other systems like hunting, where dynamically adjusting extraction to availability increases the sustainability of wild game populations (Guthery 2002, Hunter and Runge 2004).

A common objective of managers is usually to maintain or increase current harvest. Increasing availability of the resource to increase harvest can be achieved by improving natural conditions for population productivity and survival. However, in recent times managed systems are increasingly relying on the use of external inputs (Jackson et al. 2009) rather than on maintaining naturally renewable resources. In the case of harvested animal populations, an example of this is the artificial increase of resource availability through (re)stocking, an increasingly used management technique that may entail environmental costs (Laikre et al. 2010).

The red-legged partridge (*Alectoris rufa*) is a farmland game bird from southwest Europe with most of its global population located in Spain (Blanco-Aguiar et al. 2003). In addition to being a primary prey source for many Iberian predators (Calderón 1977, Herranz 2000, Duarte and Vargas 2001), this species comprises 23% of all the small game animals harvested in Spain, a proportion only surpassed by the European wild rabbit (*Oryctolagus cunniculus*). Indeed, 62% of the money paid directly for small game corresponds to both of these species (Ministerio de Medio Ambiente y Medio Rural y Marino [MARM] 2006). Despite its ecological and economic importance, wild populations of red-legged partridge have declined sharply since the 1970s for reasons associated with changes in agricultural practices and overhunting (Aebischer and Potts 1994, Aebischer and Lucio 1997, Rocamora and Yeatman-Berthelot 1999, Blanco-Aguiar 2007, Casas and Viñuela 2010). Annual harvest in Spain decreased from approximately 3.5 to 4 million partridges in the 1970s and 1980s to 2–2.5 million in the early 1990s (Blanco-Aguiar 2007). Interestingly, annual harvest from the 2000s increased again to the current level of 3.3–3.5 million partridges (MARM 2010), probably because of widespread releases of farm-bred partridges (Blanco-Aguiar et al. 2012).

In the second half of the 20th century, the number of hunters in Spain doubled and the philosophy underpinning hunting activities changed from self-sufficiency or simple family entertainment to a profitable business (Martínez et al. 2002, Martínez-Garrido 2009). Concurrent with its population decline and rising economic interest, the use of farm-bred birds to supplement wild populations of red-legged partridges started in the late 1970s and has exponentially increased ever since (Angulo 2003, González-Redondo 2004, Blanco-Aguiar et al. 2008, Ríos-Saldaña 2010). The amount of farm-bred partridges released annually is not precisely known, but estimations range between 3 million and 6 million (Delibes 1992, Pérez-Pérez 1992, Garrido 2002, Martínez et al. 2002, González-Redondo et al. 2010), a figure comparable to the current annual harvest (MARM 2010). Generally, if hunting estates release farm-bred partridges, they have to do so within restrictions on timing (no later than 2 weeks prior to the start of the hunting season in Oct) and numbers. However, regulations have been recently approved (e.g., Dirección General de Conservación del Medio Natural 1993, Consejería de Agricultura y Medio Ambiente 1996) allowing certain estates (administratively labeled as intensive) to have much fewer legal restrictions in relation to number or timing of farm-bred partridge releases. In these types of estates, large numbers of partridges (>2,000/km^2^ on average) are released annually, and throughout the whole hunting season (Ríos-Saldaña 2010; Authors, unpublished data). Intensive estates are relatively scarce (3% of all hunting estates in 2006; MARM 2006, Ríos-Saldaña 2010), but there is an increasing demand for this label.

Releases of farm-bred birds as a management tool is highly controversial among hunters, managers, and conservationists, both in Spain (Martínez et al. 2002, Gortázar et al. 2006) and elsewhere (Leopold 1944). In the case of partridges, this is because of perceived (and increasingly documented) negative effects of releases on wild red-legged partridge populations due to disease spread, changes in population genetic pool, reduction in fitness, or overhunting (Blanco-Aguiar et al. 2008, Sokos et al. 2008, Villanúa et al. 2008, Barbanera et al. 2010, Casas et al. 2012). Thus, releases could positively affect harvest by temporarily increasing partridge availability, but negative effects through reducing the survival of wild stock partridges could also be expected (Gortázar et al. 2000, Gortázar et al. 2006). Understanding the factors affecting harvest and the relationship between releases and harvest is essential to optimizing management and to assessing if the use of farm-bred partridges is having positive effects that may compensate its costs (either monetary for individual managers or ecological for the environment).

We explored the relationship between harvest and partridge availability (from wild and released birds), to evaluate whether releases have a noticeable effect on annual harvest numbers. We discussed the importance of assessing the effectiveness of management techniques to assist managers in avoiding any negative ecological effects.

## STUDY AREA

We worked in central Spain, one of the main regions for small-game hunting in Spain (Ríos-Saldaña 2010). Hunting is allowed in 89% of central Spain (Ríos-Saldaña 2010), and hunting estates are either managed by the government (13%) or privately (87%), the latter by either individuals or hunting societies. If managed privately, they may be commercial venues (the purpose of the estate is to sell hunting days to hunting customers). In any case, land management decisions are often made separately from game management decisions, as the land itself rarely belongs to the owner of the hunting rights.

**Figure 1:**
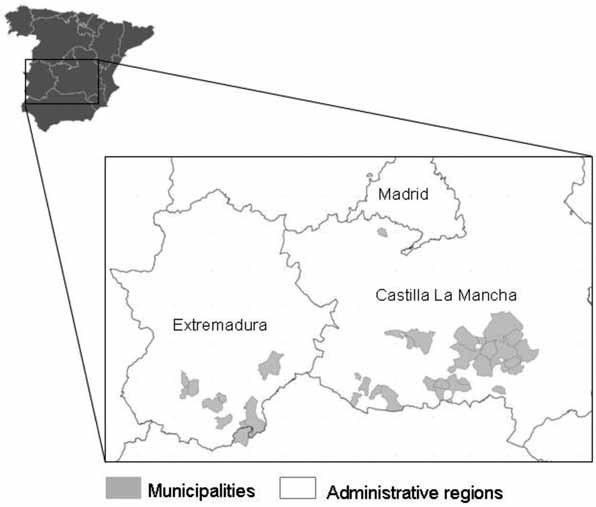
Municipalities (light gray) where we studied Harvest of red-legged partridge from 2005 to 2009 within hunting estates and their situation in peninsular Spain (top left).

We studied 50 hunting estates (all of them managed privately; Fig. 1). The total area of studied estates (1,945.87 km^2^) covered 22% of the municipalities in the study area. Hunting estate area ranged from 2 km^2^ to 280 km^2^. Most were relatively small; 22% were ≤5 km^2^, 40% had an area between 5 km^2^ and 30 km^2^, and only 6% were ≥100 km^2^. Only 6 of the 50 studied estates (amounting to 12% of the sample) were intensive. Intensive estates were those legally labeled as such, which allowed them to have few numerical and temporal restrictions on releases of farm-bred birds, whereas supplementation of artificially raised birds in non-intensive estates, if it happened, was usually more limited. As intensive estates represented only 3% of all estates available in the area, our sample was positively biased towards intensive estates.

## METHODS

To determine if harvest was related to the availability of farm-bred birds or to wild population densities, we also took into account variables of hunting pressure and habitat, because they may potentially affect this relationship. Harvest may be associated with hunting pressure, as the longer the time people are allowed to hunt in a given estate, the larger the harvest, assuming a constant intention to hunt (Palmer et al. 2002). Additionally, habitat variability between estates may have an effect on harvest irrespective of game availability or hunting pressure (e.g., by reducing the area where hunting is possible or the visibility of birds). Thus, we considered partridge abundance, release intensity, hunting pressure, and habitat simultaneously in our analyses.

### Management and Hunting Data

We interviewed managers from each study estate. Through the interviews, we obtained data on area, red-legged partridge annual harvest, farm-bred partridge annual releases, hunting pressure, and possession of an intensive hunting estate license. We calculated mean partridge harvest (harvest) as the total annual harvest in the estate divided by its area (km^2^). We conducted interviews in August 2005 (22 interviews), March 2008 (1), June 2008 (5), September 2008 (3), February 2009 (1), March 2009 (3), April 2009 (10), May 2009 (4), and February 2010 (1). When information for several years existed, we used the harvest during the game season previous to the interview, which was usually the year prior to the field survey (see below). We assumed that between-estate variability in harvest was greater than among-year variability for a given estate, and thus that our data from just 1 game season characterized the level of annual harvest for each estate. We checked this last assumption for 21 estates for which we had information on harvest for different number of years (mean ± SD = 6.71 ± 3.74 yr) and obtained a repeatability value (Lessells and Boag 1987) of 0.99, showing that harvest adequately represented harvest variability among estates.

We calculated partridges released as the number of farm-bred partridges released the year prior to the interview in each estate divided by its area (km^2^). We checked again if this variable was representative of estate release intensity for an average year with data for 47 estates (mean ± SD = 10.55 ± 2.54 yr), and obtained a repeatability value of 0.87. We also categorized each estate as intensive or non-intensive, according to whether they had the administrative category or not (variable: intensive).

We calculated hunting pressure as the product of mean number of hunters per day by the number of hunting days in the estate during the hunting season, divided by the estate area. Three main hunting methods are used in central Spain: 1) walked-up shooting, where hunters go walking alone or in small groups, with or without dogs, and shoot the game species they find along the walk; 2) driven shooting, where partridges are driven towards concealed and stationary hunters by teams of beaters; and 3) hunting with decoy, where the hunter remains hidden and shoots the wild partridges when they approach the decoy (occurring only between Jan and Mar). Walked-up shooting was the prevalent method in non-intensive estates; 86% of 44 non-intensive estates offered this method, whereas only 19% offered driven shooting days (see also Table 1). In contrast, 100% of intensive estates offered driven shooting, and 96% of all harvest occurred through driven shooting. Hunting with decoy was less common in general than the other 2 methods (Buenestado et al. 2009), and did not occur at all in 55% of our non-intensive and 34% of our intensive estates. Given that the different hunting methods may have a completely different ratio between time spent hunting and success, we measured hunting pressure separately for each hunting method.

**Table 1:** Average (±SD) values for red-legged partridge management and hunting variables in our sample taken in central Spain, 2005–2009, calculated for non-intensive and intensive estates separately

	Non-intensive	Intensive
Partridges released (number/km^2^)	13.49 ± 31.78	2,672.91 ± 2,022.94
Driven shooting pressure (hunters/season/km^2^)	0.01 ± 0.03	0.12 ± 0.09
Walked-up shooting pressure (hunters/season/km^2^)	0.13 ± 0.12	0.03 ± 0.03
Decoy shooting pressure (hunters/season/km^2^)	0.03 ± 0.08	0.04 ± 0.03
Partridge abundance index (number/observation point)	1.96 ± 3.18	1.61 ± 1.19
Harvest (number/km^2^)	33.12 ± 34.06	1,535.15 ± 1,015.09
Farmland (%)	47 ± 31	47 ± 33
Mediterranean shrubland (%)	24 ± 25	38 ± 29
Dehesa (%)	5 ± 10	3 ± 3
Woodland (%)	9 ± 24	3 ± 6
Grasslands (%)	11 ± 15	7 ± 5

### Partridge Abundance Data

We calculated a summer partridge abundance index using point count transects (Ralph and Scott 1981, Bibby et al. 1992) in each of the 50 hunting estates. Point count transects are widely used for bird population monitoring in Europe and North America, and they are considered particularly useful for red-legged partridge when the objective is a large-scale census (Onrubia 1998). Observers drove along transects, stopping every 700–750 m (exact point depending on visibility of the surrounding area). The number of points assessed in each estate was 69.6 ± 64.1 (range: 4–425 points), depending on estate area. We aimed to sample transects covering the whole of the estate, or, when they were too big, a representative area of the estate stratifying by habitat. On each point, we recorded partridge numbers and locations for 10 minutes. Observations took place in early morning (from sunrise to approx. 3 hr later) and in the evenings (last 3–4 hr of sunlight), avoiding the hottest central hours when activity was lowest. We also suspended observations during rain or when conditions were too windy. We estimated distances from partridges to observer using intervals of 50 m.

We selected survey dates to coincide with the time when most of the cereal had been harvested (in order to maximize partridge visibility), but before farm-bred partridge releases occurred (or at least before they were widespread). In non-intensive estates, releases usually took place as near as possible to the opening of the hunting season (i.e., in or after Aug). We surveyed 22 estates from 16 June to 31 July in 2006, 9 estates from 17 July to 13 August in 2008, and 19 of them from 16 June to 12 August in 2009. Furthermore, we checked with game managers or gamekeepers that partridges had not been released before the census whenever possible. Thus, we have reasonable confidence that our census must reflect abundance of wild partridges, including any possible survivors of releases from the previous hunting season. Available scientific information indicates that overwinter survival of released partridges is low (Gortázar et al. 2002, Alonso et al. 2005, Gaudioso et al. 2011, Casas et al. 2012).

We calculated a partridge abundance index as the sum of recorded partridges within a radius of 300 m at each observation point, divided by the number of observation points monitored in each estate. We did not specifically evaluate detection probability, and therefore we did not calculate population density (Bibby et al. 1992). However, this method provided comparable data between areas of relative abundance estimates. We used a 300-m radius for the index because 1) taking into account distance between observation points, a greater than 300-m radius would not confidently avoid counting the same animal twice; and 2) using much smaller radii, we had a much greater proportion of points with zero observations, which could potentially increase the error. In any case, we found strong correlations between estimates for each hunting estate calculated using each of 3 possible radii (300 m, 250 m, or 200 m); *r* coefficients ranged between 0.996 and 0.999 for 2 × 2 correlations of the 3 different estimates for each estate (*n* = 50).

### Habitat Data

We recorded the estimated percentage of each habitat type within a radius of 100 m at each observation point, during the bird surveys at each hunting estate. We defined 7 habitat categories (Table 1) with functional and management meaning for red-legged partridge: 1) arable farmland (mostly cultivated with winter cereal or left in annual fallow and usually ploughed during summer or fall), 2) vineyards, 3) tree crops (mainly olive groves, secondarily almond trees, occasionally fig trees), 4) uncultivated grasslands (including fallow land >1-yr old and uncultivated areas covered by low herbaceous vegetation), 5) Mediterranean shrubland (mainly medium-height Mediterranean shrubs, most often *Cistus* sp., *Halimium* sp., *Retama sphaerocarpa*, *Rosmarinus officinalis*, with a strong component of *Quercus coccifera* and Holm oak [*Quercus ilex*], the latter sometimes achieving full tree height), 6) woodland (pine or eucalyptus plantations, secondarily poplars), and 7) dehesa (areas of sparse oak woodland, which may be cultivated or grazed underneath). A few estates contained sparse juniper (*Juniperus phoenicea*) trees, with either pasture or crops underneath. We categorized this as dehesa because it had the same structure. Other reported habitats (riparian vegetation or country houses) occurred only marginally. For analyses, we lumped arable land, vineyards, and tree crops as farmland to further simplify habitat variables and as trends in preliminary analyses were similar for the 3 variables.

### Statistical Analysis

We tested the relationship of harvest with explanatory variables (partridge abundance index, release intensity, hunting pressure, and habitat) with general linear mixed models with a normal error of the response variable and an identity link. The model included census year as a random variable, to control for the potential effect of year on the estimation of abundance. First, we considered the whole data set, included the binomial variable called intensive as an additional explanatory variable, and constructed models with different combinations of our explanatory variables. Then, considering the large difference between intensive and non-intensive estates in both release intensity and harvest (see Results Section), we repeated the analysis separately for both groups of estates to study the effect within smaller ranges of release intensity. When analyzing data from intensive estates, we used general linear models (as all censuses but 1 were completed in a single year), and only considered relevant combinations of up to 2 explanatory variables because of the small sample size (6 estates). We considered as best models those with smaller corrected Akaike's Information Criterion (AIC_*c*_; Burnham and Anderson 2002, Bolker et al. 2008). Specifically, we considered within the set of best models those within 3 AIC_*c*_ of the top ranked model. We calculated Akaike weights for all models initially considered, and used them to estimate the relative importance of each variable by summing the Akaike weights across the set of best models where that variable occurred (Burnham and Anderson 2002). We further used the set of best models to obtain model averaged parameter estimates, and standard errors for the variables. We carried out analyses with the glm, lme, dredge, and model.average R functions (R Development Core Team 2009). We checked the goodness-of-fit of the set of best models with the adjusted *R*-squared of the linear regression between predicted and observed values of each model, and with a Shapiro–Wilk (S-W) test of normality for residuals. Finally, although we built the set of best models as explained above, we pointed out the variables possibly included as uninformative parameters following Arnold (2010), that is to say, variables appearing as one additional parameter of models with lesser AIC_c_ within the group of best models.

## RESULTS

For intensive estates, numbers of birds released was 200 times larger than for non-intensive estates, but the partridge abundance index was similar (Table 1). Harvest was 46 times larger in intensive estates (Table 1). Hunting pressure was mainly through driven shooting in intensive estates, and through walked-up shooting in non-intensive estates. Decoy shooting pressure was low in both types of estates (Table 1).

When we considered all estates together, the best models explaining variation in harvest (Table 2) included 4 habitat variables (woodland, grassland, Mediterranean shrubland, and farmland) and 5 management variables: driven shooting pressure, walked-up shooting pressure, abundance index, release intensity, and possession of the intensive label. Harvest increased with driven and walked-up shooting pressure, as well as with wild and farm-bred availability, and was greater in intensive than in non-intensive estates. It was also greater in estates with greater proportions of Mediterranean shrubland and farmland, and lesser in estates with more woodland and grassland (Table 3). However, grassland and farmland appeared as one additional parameter of the top-ranking model (Table 2), meaning that they probably were uninformative variables, which was also supported by the small relative importance calculated for them (Table 3). The relationship between observed and predicted harvest was strong in all the models (*R*^2^ = 0.99, Table 2; S-W *P* ≥ 0.225).

**Table 2:** Results of models explaining variation in red-legged partridge harvest in central Spain, 2005–2009, for a) all estates, b) non-intensive estates, and c) intensive estates. For each model, we provide number of parameters (*K*), second-order Akaike's Information Criterion (AIC_*c*_), difference in AIC_*c*_ relative to the best model (ΔAIC_*c*_), Akaike weight (*w*_*i*_), log likelihood (logLik), and adjusted *R*-squared of the linear regression between predicted and observed values (*R*^2^). The table only presents those models with ΔAIC_*c*_ ≤ 3. Variables in the models include: F = farmland, MS = Mediterranean shrubland, D = dehesa, W = woodland, G = grasslands, Ab = partridge abundance index, R = partridges released, I = having intensive license, WSP = walked-up shooting pressure, PDS = driven shooting pressure, PHD = hunting with decoy pressure

F	MS	D	W	G	Ab	R	I	WSP	PDS	PHD	*K*	AIC_*c*_	ΔAIC_*c*_	*w*_*i*_	logLik	*R*^2^
a) All estates																
	x		x		x	x	x	x	x		10	533.0	0.00	0.20	−242.6	0.99
	x				x	x	x	x	x		9	534.9	1.85	0.08	−242.9	0.99
	x				x	x	x		x		8	534.9	1.93	0.08	−249.3	0.99
	x		x	x	x	x	x	x	x		11	535.1	2.13	0.07	−242.4	0.99
x	x		x		x	x	x	x	x		11	535.8	2.76	0.05	−242.6	0.99
			x		x	x	x	x	x		9	536.0	2.97	0.04	−242.6	0.99
b) Non-intensive																
	x				x			x			6	402.5	0.00	0.09	−189.9	0.62
	x			x	x			x			7	403.8	1.22	0.05	−189.9	0.62
x		x			x			x			7	404.0	1.41	0.05	−187.6	0.66
	x				x						5	404.2	1.68	0.04	−189.9	0.62
	x	x			x			x			7	404.3	1.73	0.04	−188.3	0.66
x		x			x						6	404.4	1.83	0.04	−193.7	0.63
	x		x		x			x			7	404.5	1.97	0.03	−190.9	0.62
	x				x			x	x		7	404.7	2.16	0.03	−183.8	0.62
	x			x	x						6	404.9	2.34	0.03	−195.8	0.59
	x				x			x		x	7	405.0	2.46	0.03	−185.1	0.62
	x				x	x		x			7	405.1	2.51	0.03	−191.1	0.61
	x	x			x						6	405.1	2.55	0.03	−194.3	0.62
x	x				x			x			7	405.2	2.68	0.02	−189.9	0.62
x		x	x		x			x			8	405.5	2.96	0.02	−188.6	0.66
c) Intensive^a^																
						x					3	95.0	0.00	1.00	−35.79	0.97

a Thirty-one other competing models all had ΔAIC_*c*_ > 3.00 and *w*_*i*_ < 0.001.

**Table 3:** Model averaged parameter estimates (β), standard errors, and relative variable importance (calculated as the sum of AIC weights for models containing the parameter) for the variables included in the best models explaining red-legged partridge harvest in central Spain, 2005–2009 (i.e., those with Akaike's Information Criterion differences [ΔAIC_*c*_] ≤ 3)

	β	SE	Relative variable importance
All estates			
Intercept	−23.743	14.616	
Abundance index	8.998	2.391	0.52
Intensive license	164.982	34.397	0.52
Driven shooting pressure	782.421	193.251	0.52
Partridges released	0.476	0.011	0.52
Mediterranean shrubland	0.604	0.274	0.48
Walked-up shooting pressure	138.387	64.477	0.44
Woodland	−3.266	1.609	0.36
Grassland	−0.453	0.459	0.07
Farmland	0.162	0.241	0.05
Non-intensive			
Intercept	9.714	14.487	
Abundance index	7.668	1.145	0.53
Mediterranean shrubland	0.359	0.142	0.42
Walked-up shooting pressure	57.296	29.246	0.39
Dehesa	−0.296	0.186	0.18
Farmland	−0.242	0.191	0.13
Grassland	0.303	0.239	0.08
Woodland	−0.854	0.882	0.05
Driven shooting pressure	90.149	115.791	0.03
Decoy hunting pressure	−23.864	41.212	0.03
Partridges released	0.056	0.10348	0.03
Intensive			
Intercept	211.15	130.25	
Partridges released	0.4953	0.040	1.00

**Figure 2:**
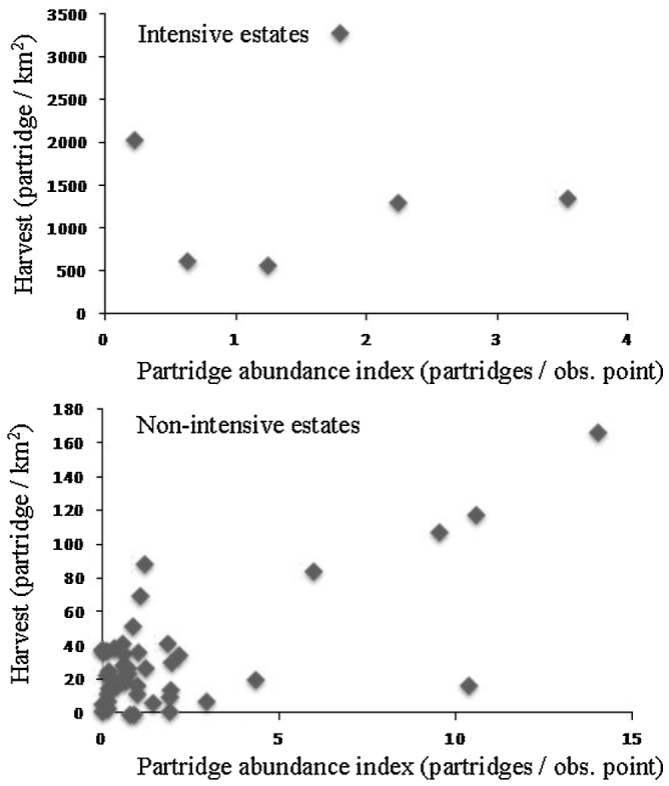
Relationship between red-legged partridge harvest and summer abundance for intensive estates (above) and excluding intensive estates (below) in central Spain, 2005–2006.

For non-intensive estates, the best models with informative parameters explaining variation in harvest included 2 management variables, partridge abundance index, and walked-up shooting pressure (Table 2), both positively related to harvest (Table 3), and 3 habitat variables (Table 2), Mediterranean shrubland positively related to harvest and farmland and dehesa negatively related (Table 3). Mediterranean shrubland was the habitat variable with greatest relative importance (Table 3). All the other management and habitat variables studied (driven shooting pressure, decoy hunting pressure, partridges released, woodland, and grassland) were included in some of the best models, but they were probably uninformative parameters, because they appeared as one additional parameter of models with lesser AIC_*c*_. The relationship between observed and predicted harvest gave an adjusted *R*-squared between 0.59 and 0.66 (Table 2; S-W *P* ≥ 0.013). The relationship between abundance estimates and harvest in non-intensive estates, although significant, was very scattered, particularly among estates with lesser abundances of birds (Fig. 2). The relationship relied on a small number of game estates with high summer bird densities. If we removed from the analyses the 5 estates with summer abundance indices ≥5, the relationship disappeared, and the only variables affecting harvest in these estates were walk-up shooting pressure and Mediterranean shrubland habitat.

**Figure 3:**
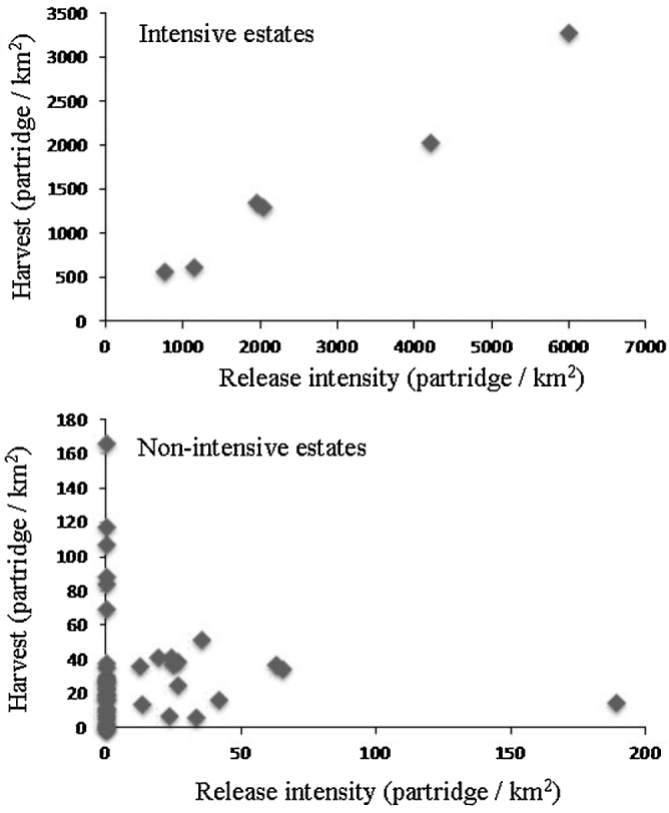
Relationship between red-legged partridge harvest and releases for intensive estates (above) and excluding intensive estates (below) in central Spain, 2005–2009.

When looking at intensive estates separately, the best model explaining variation in harvest included only 1 variable: partridges released (Tables 2 and 3). We found a linear relationship between releases and harvest in these estates, which indicated that approximately 45% of released partridges were harvested (Fig. 3, Table 3). The relationship between observed and predicted harvest was strong (*R*^2^ = 0.97, Table 2; S-W *P* = 0.686).

## DISCUSSION

Our study indicates that, in central Spain, variation between estates in red-legged partridge harvest was related to both partridge availability and hunting pressure, but with marked differences between intensive and non-intensive estates. In intensive estates, harvest was linearly dependent exclusively on release intensity. In non-intensive estates, harvest increased mainly with wild partridge densities and walked-up shooting hunting pressure, and releases only had a minor effect in one of the 14 best models. The main effect of habitat was an increase of harvest with increasing abundance of Mediterranean shrubland.

### Harvest in Intensive Estates

In intensive hunting estates, harvest was exclusively and linearly related to the number of partridges released; frequency of releases is probably adjusted by managers to short-term harvest demand, and the numbers of partridges released is adjusted to the number of hunters. This would explain the absence of effects of hunting pressure on harvest. On intensive estates, releases are usually allowed over a longer period of the year than in non-intensive estates. According to the coefficient in the model, the mean return on harvest of partridges released is around 45%, although partridge summer densities are not greater than in non-intensive estates, suggesting a loss of more than half of the released birds both before and after the shoots. This is concurrent with the high mortality of released partridges reported in other studies (Gortázar et al. 2000, Alonso et al. 2005).

In intensive estates, we found no relationship between harvest and summer partridge abundance, confirming that in these estates hunting is detached from in situ natural resource management and is approaching an industrial activity based on external inputs. From an ecological and managerial point of view, commercial activities based on natural populations or on farm-bred animals have entirely different objectives and natural resource sustainability. Rules to avoid dangers of large quantities of animals establishing in free-ranging populations or disease transmission to native wildlife should be adopted (e.g., The Wildlife Society 2012). Also an administrative separation of estates employing each type of management, not only legally (as happens now with the legal label) but also potentially in terms of taxes or commercial eco-labels would be relevant, as it would reward managers that preserve multifunctional estates by maintaining healthy wild populations whilst allowing them to compete in the market. This separation was also recommended in the conclusions from the review of Sokos et al. (2008).

### Harvest in Non-Intensive Estates

Harvest in non-intensive estates was positively related to summer partridge abundance, but the relationship relied on those few estates with greatest summer densities. In estates with moderate or low summer abundance indices, we did not find any relationship between harvest and wild partridge abundance estimates. We cannot discard that our abundance index was not sensitive enough to clearly distinguish among low abundances, and thus some noise in the relationship may come from the abundance index itself. Also, partridge releases may have been unreported by managers during the interviews in some of those 5 estates with high summer bird densities. Selling farm-bred partridges as if they were wild partridges may be a highly profitable business that, obviously, must be based on hiding release activity to the public. The increasing likelihood of this fraudulent activity when releases are more widespread has been previously mentioned (Delibes 1992). Our results show that the relationship between harvest and availability was not strong with low abundances, which was also found by Cattadori et al. (2003) studying red grouse (*Lagopus lagopus scoticus*) harvest. This suggests that either estimation of abundance made by managers in certain estates is poor, or that other criteria are used to determine harvest. For example, harvest in some partridge estates may be determined by the willingness of hunters to hunt even if populations are low, so hunting pressure may be greater than expected from wild stock abundance. This may be relevant whenever hunters lease an estate for a short time and they do not intend to renew the lease in subsequent years so the concern about long-term sustainability of hunting in that estate is low or non-existent. This also occurs when land owners or game managers do not establish any regulatory or monitoring framework for hunting pressure for the hunters hiring the hunting rights, as happened on some of the estates in our sample. Overall, underharvesting to guarantee survival of populations, and particularly overharvesting to maximize short-term yield could be happening in a proportion of the estates. Given that overharvesting is particularly dangerous for population sustainability, particular care should be taken to minimize this risk.

Harvest in non-intensive estates was positively related to walked-up shooting pressure, which suggests that estates with more hunters or more frequent hunts may overall hunt more than it should be appropriate for availability (Watson et al. 2007). It has already been suggested that at the national level an increase in the number of hunting licences (and thus hunting pressure) in the 1970s was a main factor leading to the decline in red-legged partridges at that time (Blanco-Aguiar et al. 2003). Similarly, hunting pressure has been found to be negatively associated with European wild rabbit population trends in northeastern Spain (Williams et al. 2007). Managers should look for a balance between the monetary or social benefits of increasing shooting pressure in non-intensive estates, and the effect in partridge population abundance, which also may have negative monetary and social consequences.

Furthermore, in non-intensive estates, supplementing partridges in relatively small numbers (studied range: 12–189 partridges/km^2^) had no noticeable effect on harvest. Releases in non-intensive estates may be being used to attract hunters to estates with low-density populations, but this management action seems to be inefficient. The high percentage of rapid losses of released partridges when using traditional management (Gortázar et al. 2000) probably prevents any marked increase in availability when releases are performed in small numbers. Release methodologies and wild densities differ in non-intensive estates, which could increase the variability in the relative effect of releases. Considering this general lack of effect on harvest, we were surprised that small-scale releases are frequently and increasingly used in these estates. For example, 38% of non-intensive estates in our study region declared to release partridges (Ríos-Saldaña 2010). This raises the question of the relative benefits and costs of this management technique, and for whom the releases benefit. If releases are used only to maintain hunting activities in estates with low populations of partridges, our results suggest that this action is not cost effective (e.g., Musil 2004) and should be avoided on an economical basis. Alternatively, they may be carried out to help the recovery of wild populations, but this needs careful management of releases and many failures have been recorded (Leopold 1944, Potts 1986). Releases in non-intensive estates as a tool for population reinforcement should only be allowed if the strategy also includes stopping hunts in the estate until the desired abundance is attained.

Finally, we found a relationship between habitat and harvest, which tended to be greater as the area covered by Mediterranean shrubland in the estate increased. Red-legged partridges tend to use shrubland more frequently during fall and winter (Lucio and Purroy 1987, Lucio 1991), and our results may be reflecting this seasonal pattern of habitat selection. In contrast, increasing percentages of area covered by dehesa, farmland, or woodland negatively affected harvest. Dehesa and farmland are open habitats where partridges may probably escape walking hunters more easily, whereas woodland is a habitat generally avoided by partridges (Blanco-Aguiar et al. 2003).

Our results show that partridge harvest in non-intensive estates with low abundance is mainly related to hunting pressure possibly creating a mismatch between harvest and availability in our study area. Increasing the abundance of wild red-legged partridge through techniques like habitat management (which has been suggested as an effective measure; Casas and Viñuela 2010), and improving the adjustment of harvest to availability like Lucio (1998) already recommended, is advised for partridge managers. Overall, our results lead to the questions of what is the future viability and sustainability of partridge hunting and if we may be depleting our natural capital (Costanza et al. 1997, Daily 1997, Woodworth 2006). Inaccurate or unavailable information about spatial distribution and numbers of released birds, wild contingents, harvest numbers, and the general benefit of this management technique at a large-scale does not help to answer these questions. Similarly, not including environmental costs in management may be promoting a lack of environmental efficacy and environmentally expensive management.

## MANAGEMENT IMPLICATIONS

The common practice of releasing small numbers of farm-bred partridges had little impact on annual harvest, and thus this practice is not an effective tool to sustain traditional hunting. Together with described negative effects on wild red-legged partridge populations, we predict that their use would have a negative effect on harvests of wild birds, leading to increased dependence on releases. On the other hand, massive releases in small areas are effective at increasing annual harvest, and they have a locally high social and economical effect in the short term. Government and managers need to carefully select where to locate intensive estates, and to create ways to label and segregate the estates where only wild red-legged partridges are managed from those where releases are used. This would allow hunters to use restocking as additional criteria to select their preferred estates (currently, trustworthy guarantee to do so does not exist), and would reduce ecological costs of management decisions. Moreover, we strongly encourage authorities in charge of game preservation and game managers to improve game information recording systems, hunting laws, and management techniques, for the sake of future exploitation of a unique game resource that may be currently globally endangered.
